# The impact of telework on absenteeism, presenteeism, and return to work among workers with health conditions: a scoping review

**DOI:** 10.3389/fpubh.2025.1655200

**Published:** 2025-09-09

**Authors:** Julien Ducas, Catherine Daneau, Salma Bouqartacha, Alexandra Lecours, Jacques Abboud, Andrée-Anne Marchand, Martin Descarreaux

**Affiliations:** ^1^Department of Anatomy, Université du Québec à Trois-Rivières, Trois-Rivières, QC, Canada; ^2^Groupe de Recherche sur les Affections Neuromusculosquelettiques (GRAN), Université du Québec à Trois-Rivières, Trois-Rivières, QC, Canada; ^3^Department of Human Kinetics, Université du Québec à Trois-Rivières, Trois-Rivières, QC, Canada; ^4^Department of Occupational Therapy, Université du Québec à Trois-Rivières, Trois-Rivières, QC, Canada; ^5^Center for Interdisciplinary Research in Rehabilitation and Social Integration, Quebec City, QC, Canada; ^6^Department of Chiropractic, Université du Québec à Trois-Rivières, Trois-Rivières, QC, Canada

**Keywords:** presenteeism, absenteeism, return to work, telework, telecommuting, remote work, sick leave, illness

## Abstract

**Introduction:**

Telework has become increasingly prominent as a flexible work arrangement, particularly since the COVID-19 pandemic. For workers managing health conditions, it may support continued employment by influencing key work-related phenomena such as absenteeism, presenteeism and return to work (RTW) process. However, current evidence on the impact of telework on the work-related outcome to manage health condition in the workplace remains limited and fragmented.

**Objective:**

This scoping review aimed to map the existing literature on the impact of telework on absenteeism, presenteeism, and RTW outcomes among adult workers with health conditions.

**Methods:**

Included studies were either qualitative, quantitative, or mixed methods, published in English or French, including adults with any physical or psychological health conditions. At least one outcome domain (absenteeism, presenteeism, or RTW) was required. Eight databases were searched from inception to May 2025: Medline, CINAHL, APA PsycINFO, Academic Search Complete, Business Source Complete, Scopus, Sociological Abstracts, and ABI/INFORM Global. Data extraction focused on study design, objectives, variables/definitions, sample size, health status, demographic characteristics, individual characteristics, organizational factors and results. Data were synthesized by the outcome domain (absenteeism, presenteeism, RTW) and stratified by study type (quantitative vs. qualitative).

**Results:**

From 4,093 records, 21 studies were included. The majority of studies suggest that telework contributes to reduced absenteeism by increasing work flexibility. Telework is also consistently associated with facilitating RTW, particularly following surgery or in the context of chronic illness, by supporting work reintegration and shortening the duration of sick leave. In contrast, findings on presenteeism are conflicting: some studies report that telework increases the likelihood of working while sick, others suggest a decrease, and some report no significant impact or conflicting results. These outcomes appear to be influenced by contextual factors, including health status, demographic variables, individual characteristics, and organizational context.

**Conclusion:**

Telework appears to offer flexibility that can reduce absenteeism and facilitate RTW. However, its impact on presenteeism is less consistent and may even encourage working while sick if not properly supervised. Future studies should examine which policies most effectively maximize the benefits of telework while minimizing potential drawbacks.

## Introduction

Workers living with health conditions, whether acute, recurrent, or chronic, represent an important portion of the labor force (1). These conditions can range from acute illnesses such as the common cold or fever, to chronic or recurrent conditions such as musculoskeletal disorders, cardiovascular disease, diabetes, depression, anxiety, or cancer (2, 3). Adults contract an average of 2 to 3 colds per year, often accompanied by fever, which can temporarily impair their work capacity (3, 4). Moreover, in Canada, nearly 44% of adults aged 20 years and older report having at least one of 10 common chronic conditions, such as osteoporosis, arthritis, anxiety disorders or cancer, which can limit their ability to work due to injury-related impairment or post-surgical recovery (2).

As a result of their condition, workers may experience absenteeism, presenteeism, or challenges related to returning to work (RTW). These three work-related outcomes significantly impact employee well-being and organizational productivity (5, 6). Sickness absenteeism refers to an employee’s failure to attend work as scheduled due to sickness (7, 8), while presenteeism occurs when employees attend work despite being unwell, leading to reduced productivity and performance (8, 9). RTW process involves the transition back to work following a health-related absence (10). A successful RTW is often a gradual and supported process that considers the worker’s functional capacity and workplace accommodations (10, 11).

Absenteeism, presenteeism, and challenges related to RTW collectively have a substantial economic burden. Studies have estimated that in the United States, employees with cancer, chronic lung disease, cardiometabolic disorders, pain or depression lose a median of 33.9 work hours annually due to absenteeism, with associated annual productivity losses ranging from $100 to $10,000 per worker (12). In Canada, the Conference Board of Canada estimated that the direct cost of absenteeism to the national economy was $16.6 billion in 2012 (13). Presenteeism is hypothesized to result in even greater productivity losses and costs, as employees who work while unwell are less effective, contributing fewer effective working hours overall (14, 15). RTW process following a health-related absence presents its own challenges and economic implications, as delayed or unsupported RTW can prolong work disability and increase indirect costs (16).

Absenteeism, presenteeism and RTW processes are influenced by a variety of factors, including health status, demographic characteristics, individual characteristics, and organizational factors (10, 17). Among these, telework, defined as working outside the employer’s premises using modern information and communication technology, has emerged as a key organizational factor in the 21st century (18). The rise of telework, especially during the COVID-19 pandemic, has increased interest in its influence on absenteeism, presenteeism and RTW process as it provides employees with greater flexibility to manage their work while dealing with illness (19, 20). Sickness absenteeism generally declined during the pandemic among teleworkers, possibly due to increased flexibility and a reduced risk of exposure to workplace-related health hazards (21). In contrast, sickness presenteeism was reported to increase during the mandatory transition to remote work, possibly due to factors such as the blurred boundaries between work and personal life, and the ability to continue to work from home even while unwell (21, 22). Furthermore, it has been suggested that telework can support RTW processes and improve employment opportunities for individuals with health conditions by removing mobility barriers, reducing employer bias, enabling knowledge-based tasks, providing flexible support, and offering career development opportunities (23, 24). However, telework can lead to some unhealthy behavior. In addition to blurring the boundaries between work and personal life and increasing presenteeism, it may contribute to social and professional isolation, reduce access to informal support, limit the availability of adequate equipment or accommodations, increase sedentary behavior, increase feelings of resentment from colleagues, and restrict opportunities for career advancement or promotion (21, 25, 26). Therefore, telework should be considered as one of many possible accommodations with promising outcomes, though not without limitations.

While the impact of telework on presenteeism and absenteeism during the COVID-19 pandemic has been studied (20), its effects on individuals with health conditions remain unclear in contexts where telework is not mandated by employers or governments. This distinction is important for assessing the viability of telework as a long-term workplace accommodation, not only for managing absenteeism and presenteeism, but also for facilitating RTW processes for workers with health conditions.

Therefore, this scoping review aims to describe the impact of telework on absenteeism, presenteeism and RTW process among adult workers with health conditions.

## Methods

A scoping review, as described by Peters (27), was conducted to identify knowledge gaps, map existing literature and examine research practices (28). The PRISMA extension for scoping reviews checklist was used to ensure all necessary elements were included (29).

### Research question

What is the impact of telework on absenteeism, presenteeism, and RTW among adult workers living with health conditions?

### Search strategy

The database search was carried out with the assistance of the university librarian, who reviewed the selected databases and fine-tuned the search strategy, i.e., the choice of keywords and their search sequence. The search in the databases was carried out from their inception up to May 2025. The following databases were searched: Medline, Cumulative Index to Nursing and Allied Health Literature (CINAHL), APA PsycINFO, Academic Search Complete, Business Source Complete (BSC), Scopus, Sociological Abstract and ABI/INFORM Global. They were chosen for their relevance to the field of knowledge covered by our research question, namely health, management, rehabilitation and social sciences. The search strategy was launched in the databases according to the combination of MeSH or non-MeSH terms as follows: (telework) AND [(RTW) OR (absenteeism OR presenteeism)]. The complete search strategy for all databases can be found in the Supplementary material 1 and an example of search strategy in MEDLINE can be found in Table 1. EndNote 21.4 (Clarivate Analytics) was used to remove duplicates and article sorting was performed in Covidence.

**Table 1:** Example of search strategy in MEDLINE.

Concepts	Search terms
Concept 1 *Telework*	AB (“Remote work” OR Telecommut* OR Telehomework* OR Telework* OR “Home-based office” OR “Home-based telecommut*” OR “Home-based work” OR “Home work*” OR Home-work* OR “Virtual office” OR “Virtual work” OR Home-office OR Home office OR “Work* n3 home”) OR TI (“Remote work” OR Telecommut* OR Telehomework* OR Telework* OR “Home-based office” OR “Home-based telecommut*” OR “Home-based work” OR “Home work*” OR Home-work* OR “Virtual office” OR “Virtual work” OR Home-office OR Home office OR “Work* n3 home”) OR MH (“Teleworking”)
Concept 2 *RTW*	AB (Return-to-work OR RTW OR “Return to work” OR “Back to work transition” OR “Job re-entry” OR “Sick leave” OR “Sick day” OR “Health-related absence” OR “Medical leave” OR “Absence due to illness”) OR TI (Return-to-work OR RTW OR “Return to work” OR “Back to work transition” OR “Job re-entry” OR “Sick leave” OR “Sick day” OR “Health-related absence” OR “Medical leave” OR “Absence due to illness”) OR MH (“Return to Work” OR “Sick Leave”)
Concept 3 *Absenteeism or presenteeism*	AB (Absenteeism OR Non-attendance OR Absence OR Presenteeism OR “Over attendance” OR “Working while sick”) OR TI (Absenteeism OR Non-attendance OR Absence OR Presenteeism OR “Over attendance” OR “Working while sick”) OR MH (“Absenteeism” OR “Presenteeism”)
Combination of concepts	Concept 1 AND (concept 2 OR concept 3)

Limits: None; Last search: 8/05/2025. AB = abstract, TI = title, MH = MeSH.

### Inclusion and exclusion criteria

To be considered for inclusion, articles had to be written in English or French, and had to use a qualitative, quantitative or mixed methods design. Included participants had to be 18 years of age or older and report at least one physical or psychological health issue that could impact work-related phenomena, ranging from common cold to chronic pain. The articles had to include at least one outcome related to: (1) absenteeism, (2) presenteeism, or (3) RTW. Articles were excluded if telework was mandated by employers or government policies (e.g., during a pandemic). The following types of articles were excluded: literature reviews and meta-analyses, case studies, case series, unpublished manuscripts, dissertations and theses, government reports, books or book chapters, protocol, opinion and commentary, conference abstracts, replies to editors, letters, and any non-scientific documents. These types of articles or documents were not included, as they either lacked scientific value in the context of the present scoping review, or because they did not provide original data needed to answer the research question. Literature reviews and meta-analyses were specifically excluded to avoid duplication of information and because original results were required.

### Evidence screening and selection

Database searching was carried out by one team member (C. D.). Screening of titles and abstracts was carried out independently by two team members (C. D. and J. D.). Studies were classified in the Covidence software according to the following three choices: no (if the article did not correspond to our inclusion criteria), maybe (if there was insufficient information in the title and abstract to include the article, in case of doubt this choice was made) and yes (if the article met all inclusion criteria and no exclusion criteria). In the event of conflicts between the two independent reviewers, the evaluation of conflicting articles was carried out by three other team members (A-A. M., J. A. and M. D.). All articles classified as “maybe” and “yes” were fully read by two team members (C. D. and J. D.) to determine the total number of articles eligible for the present scoping review. After the full-text screening phase, in the event of disagreement, three other team members (A-A. M., J. A. and M. D.) were consulted to reach a consensus on the total number of articles to be included.

### Data extraction

Three team members (C. D., J. D. and S. B.) extracted data relevant to the scoping review from 21 studies: authors, publication year, study design, objectives, variables/definitions, sample size, health status, demographic characteristics, individual characteristics, organizational factors and results. Contextual factors, such as health, demographic, individual, and organizational variables, were specifically extracted as they may explain or directly influence the effects of telework on presenteeism, absenteeism, and RTW, see Supplementary material 2. The data extraction table was piloted and validated prior to use, and each data item was extracted by at least two team members.

Extracted data were synthesized by grouping studies according to the outcomes examined (presenteeism, absenteeism, and RTW). Subsequently, subgroups were created based on the type of results reported, quantitative or qualitative.

## Results

### Study contexts

**Figure 1:**
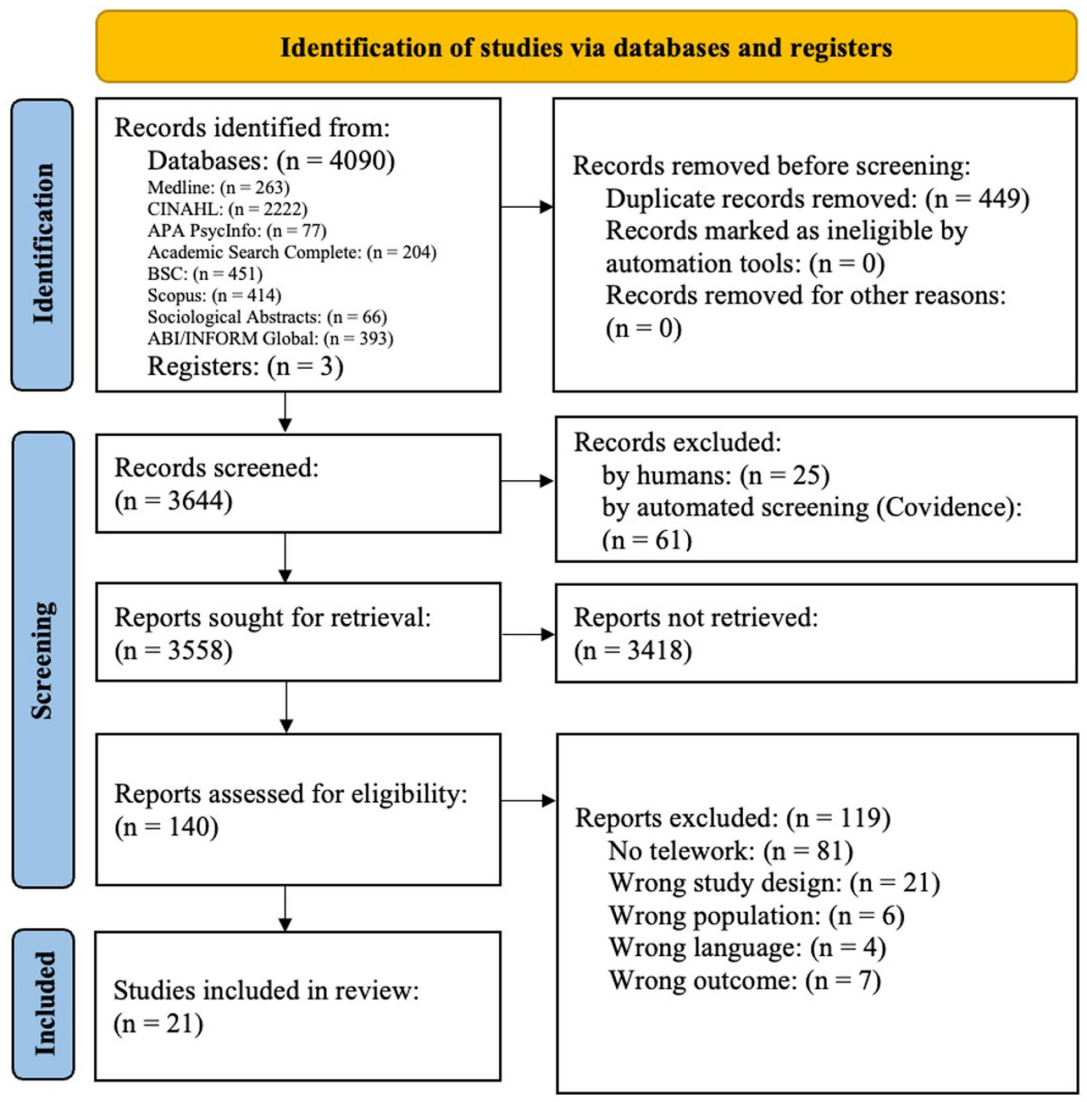
PRISMA flow chart illustrating the study selection process.

A total of 4,093 articles were identified and 21 studies were included in this scoping review, including 12 quantitative studies (30–41), 7 qualitative studies (42–48), and 2 mixed-methods studies (49, 50). A flow chart of the literature search results is shown in Figure 1. The quantitative studies were conducted in Canada (30), the United States (39), Japan (40), Germany (31), Finland (32), Spain (33), Norway (34), the Netherlands (35, 41), Sweden (36), New England (37) and Belgium (38). These studies primarily assessed the impact or associations between telework and absenteeism, presenteeism, or RTW experiences.

The qualitative studies were conducted in Canada (47, 48), Belgium (42), the United States (43), Sweden (44), the United Kingdom (45), and the Netherlands (46). These studies qualitatively assessed employees’ experiences and perceptions of telework in relation to absenteeism, presenteeism, and RTW experiences. Additionally, two mixed-method studies from Canada (50) and the United States (49) combined the two approaches. Additional details on the studies’ methodology and samples can be found in Supplementary material 2.

### Telework and sickness absenteeism

#### Measurement of absenteeism

Of the 21 studies included, 7 (33%) assessed absenteeism. Among these, 6 studies (30, 34, 38, 39, 42, 43) used a self-certified sickness absence measure, generally consisting of questions about whether the participant had taken such leave within a defined period and how many episodes had occurred. One study assessed absenteeism using data derived from a database (36).

#### Impact of, or association between telework and absenteeism

**Figure 2:**
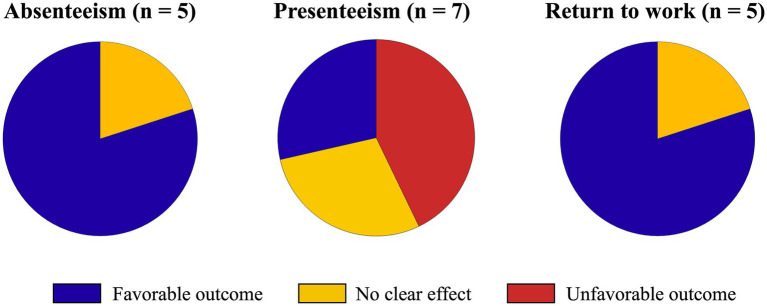
Summary of quantitative findings (including associative, causal, and descriptive statistics) on the impact of telework on absenteeism, presenteeism, and return-to-work outcomes. The chart illustrates the proportion of studies reporting favorable (e.g., decrease absenteeism or presenteeism or facilitate return to work), no clear effect, or unfavorable effects of telework on outcomes (e.g., increase absenteeism or presenteeism or hinder return to work).

Of the 7 studies that assessed absenteeism, 5 used a quantitative design. Overall, the findings were consistent, with most studies (*n* = 4, 80%) suggesting that telework can reduce absenteeism (34, 36, 38, 39). Only one study (*n* = 1, 20%) found no significant effect (30) (Figure 2).

For instance, Borge, Johannessen (34) found that telework significantly reduced the odds of self-certified sickness absence (odds ratio (OR): 0.86, 95% confidence interval (CI) 0.74–0.99), although it had no effect on the number of absence episodes (incidence rate ratio (IRR): 1.01, 95% CI 0.89–1.15) (34). Similarly, a study by Helgesson, Gustafsson (36) reported that individuals with mental health disorders (such as depression, anxiety, and stress) were more likely to take long-term sick leave if they lacked access to telework, as those who did not have telework options were 1.37 times more likely to experience over 14 days of sickness absence within a three-year follow-up period (relative risk (RR): 1.37, 95% CI 1.13–1.66) compared to those with the option (36). Van Doninck, Van Doninck (38) studied workers recovering from arthroscopic partial meniscectomy and found that teleworking workers took significantly fewer sick leave days (mean 10 days, 95% CI: 1.79–18.41) compared to non-teleworking workers, who took an average of 35 sick leave days from the date of surgery to their RTW (95% CI: 30.13–40.24; *p* < 0.001) (38). Finally, Ahmed, Kim (39) reported that among adults with influenza-like symptoms, the proportion who did not work at all was significantly lower among those with access to telework (28%) compared to those without access (41%; *p* < 0.001). In fact, adults with access to telework took significantly less time off due to illness compared to those without access (mean 0.80 days vs. 1.10; *p* < 0.001) (39).

However, not all the included studies supported the beneficial effect of telework on absenteeism. Gignac, Cao (30) investigated employees with osteoarthritis and inflammatory arthritis and found no significant difference in absenteeism among employees who needed and used telework, those who needed it but did not use it, and those who did not need telework (30).

#### Qualitative findings on telework and absenteeism

Of the 7 studies that assessed absenteeism, 2 used a qualitative design, which found that telework may help reduce absenteeism by providing employees with greater flexibility to manage their health (42, 43).

For instance, Coenen, Schmickler (42) found that employees with inflammatory bowel disease were able to better manage their health complaints with the flexibility of telework, which allowed them to control their workflow and take necessary breaks, leading to fewer sick days (42). Similarly, Frolick, Wilkes (43) reported in their study that teleworkers (health condition unknown) had lower sickness absenteeism rates compared to the general workforce, as the flexibility to adjust schedules for medical appointments and alternate between work and rest when feeling unwell contributed to fewer sick days (43).

### Telework and sickness presenteeism

#### Measurement of presenteeism

Of the 21 studies included, 8 (38%) assessed presenteeism. Several tools were used to measure sickness presenteeism. One study used a modified version of the Hagerbaumer Presenteeism Scale (omitted one item) to assess behaviors such as working despite severe symptoms and dragging oneself to work during illness (35). Two studies measured sickness presenteeism propensity by calculating the ratio of presenteeism days (days worked while sick) to the total days of sickness (presenteeism + sickness absence), with scores ranging from 0 (no presenteeism) to 1 (complete presenteeism) (31, 33). One study used the Work Limitations Questionnaire (WLQ), evaluating work limitations due to illness, with higher scores indicating greater work limitations (30). Moreover, one study used the Work Functioning Impairment Scale (40). Furthermore, three studies measured sickness presenteeism using self-reported measures by asking participants if they worked while sick over a specific time frame and the total number of days worked during illness (31, 37, 39). Finally, one study assessed presenteeism through semi-structured interviews; however, the specific questions asked could not be retrieved (43).

#### Impact of, or association between telework and presenteeism

Of the 8 studies that assessed presenteeism, 7 used a quantitative design. Findings on the relationship between telework and presenteeism were conflicting. Three studies (43%) found that telework increases sickness presenteeism (31, 33, 39), while two others (29%) found a reduction (35, 37), and two (29%) reported no significant effect (30) or conflicting results (40) (Figure 2).

For instance, Goñi-Legaz, Núñez (33) found a positive association (*p* < 0.001) between home-based telework and the propensity for presenteeism (health condition unknown) (33). Similarly, Steidelmüller, Meyer (31) found that home-based telework, regardless of its frequency (occasional, monthly, weekly, or daily), was significantly associated with an increase in sickness presenteeism prevalence and propensity compared to individuals who never teleworked. These associations remained significant even after adjusting for individual and job-related factors, including health status (chronic illness), employment characteristics (part-time work and computer use), work engagement, work intensity, working during free time, organizational factors (telework regulations, structure, and climate), work–family conflict, and job autonomy. The only effect that disappeared was for occasional teleworkers when the model accounted for working during free time. Sickness presenteeism prevalence and propensity increased with telework frequency. For instance, occasional teleworkers had an increase of 6.12% (*p* < 0.001) in sickness presenteeism prevalence and 3.32% (*p* < 0.001) in sickness presenteeism propensity compared to non-teleworkers, while daily teleworkers had an increase of 10.69% (*p* < 0.001) in sickness presenteeism prevalence and 14.74% (*p* < 0.001) in sickness presenteeism propensity compared with non-teleworker. Moreover, intraclass correlations showed that country differences accounted for 8.62% of the variation in sickness presenteeism prevalence and 6.12% in sickness presenteeism propensity, indicating that the association between home-based telework and presenteeism is more likely explained by individual characteristics and organizational differences within countries, which themselves may be influenced by country-specific labor practices, rather than country differences alone. Regarding sex differences, males showed slightly higher sickness presenteeism prevalence rates than females, but females had higher sickness presenteeism days. For sickness presenteeism propensity, males generally scored higher, except among daily teleworkers, where both sexes showed nearly 15% (31). Moreover, Ahmed, Kim (39) found that individuals with influenza-like symptoms with access to telework were significantly more likely to work during the first 3 days of illness (adjusted ratio: 1.25; 95% CI: 1.07–1.46). Overall, individuals with access to telework worked more days while sick compared to those without access (mean 1.46 vs. 1.09 days; *p* < 0.001), which was attributable to more days spent teleworking, as there was no significant difference in the number of days worked at the usual workplace while ill (39).

In contrast, Rousculp, Johnston (37) found that telework reduces sickness presenteeism in individuals with influenza-like illness. Among the flexible sick leave policies examined, such as adjusting working hours, taking time off without pay, and working from home, the only one that was significantly associated with sickness presenteeism prevalence was the ability to work from home after adjusting for employee and workplace characteristics. The predicted probability of attending work while experiencing the most severe influenza-like illness symptoms was 71.9% for employees without remote work access, compared to 57.4% for those with the option [14.5% point reduction (*p* = 0.006)]. Furthermore, employees who could work from home were 29.7% less likely to attend the worksite during severe symptoms (IRR = 0.703, *p* = 0.026) (37). Similarly, Cook and van den Hoek (35) found that, in a sample experiencing period pain, a higher number of telework days per week was negatively associated (ß = −0.10) with period pain presenteeism (*p* = 0.01). However, this association was only significant when considering the entire sample, which included individuals with various pelvic medical conditions, but not when examining those with endometriosis alone, those without a diagnosed condition, or both groups combined. Moreover, when illness disclosure to a leader was included as a mediator in the analysis, the association between telework and period pain presenteeism was no longer significant (35).

Finally, Gignac, Cao (30) reported no significant differences in presenteeism among employees with osteoarthritis and inflammatory arthritis, regardless of whether they needed and used telework, needed it but did not use it, or did not need telework (30). Similarly, Takasaki (40) found no significant differences in presenteeism across sex (male vs. female) and telework status (full-time teleworkers >70% vs. non-full teleworkers <70%) for individuals with pain, except between male non-full teleworkers and female full-time teleworkers (*p* = 0.029), with female full-time teleworkers reporting higher levels of presenteeism (40).

#### Qualitative findings on telework and presenteeism

Of the 8 studies that assessed presenteeism, 1 used a qualitative design. Frolick, Wilkes (43) found that many individuals using telework reported working productively despite being ill. They indicated that without the option to work from home, they would have taken sick leave on certain occasions. Participants were also more likely to work at least part of the day while unwell, as telework allowed them to alternate between work and rest, or to stop entirely when feeling overwhelmed (43).

### Telework and RTW

#### Measurement of RTW

Of the 21 studies included, 10 (48%) assessed RTW. Three quantitative studies (32, 38, 41), five qualitative studies (44–48), and two mixed methods studies (49, 50) assessed which accommodations or workplace conditions facilitated, or could facilitate the RTW process following a leave of absence due to a health condition.

#### Impact of, or association between telework and RTW

Among the 5 studies with quantitative data (3 quantitative, 2 mixed methods), most studies assessing RTW (*n* = 4, 80%) reported that telework facilitated the process (32, 38, 41, 49), while one study (20%) found no direct association but identified an indirect relationship (50) (Figure 2).

For instance, Kangas, Soini (32) found that, based on descriptive percentages, telework was the most used work arrangement among task modifications, increased breaks, flexible working hours, colleague support, workplace adjustments and improved commuting conditions to facilitate RTW after hip or knee arthroplasty, with 20% of participants using it (32). Moreover, Varekamp and Van Dijk (41) found that remote work was a common workplace accommodation for individuals with chronic diseases. Among 122 participants, 15% were already working from home, with 6% considering it their preferred accommodation over others such as reduced hours, a slower work pace, and assistance from others. Additionally, 30% wished to work remotely but had not yet done so, while 50% neither worked from home nor preferred it (41). Similarly, Tremblay (49) reported that among workers with bipolar disorder, remote work was a common accommodation, with 42% of them being allowed to work from home (49). Moreover, Van Doninck, Van Doninck (38) reported that telework was significantly associated with early RTW (*p* ≤ 0.001). Multivariate analysis identified telework as the only independent variable significantly associated with a faster RTW, explaining 22% of the variance after adjusting for the physical demands of work and employment status. Additionally, 45% of non-teleworkers believed that their RTW duration would have been shorter if telework had been available (38).

In contrast, Negrini, Corbière (50) found that, among workers aged 45 and older, with psychological or physical work-related health conditions, workplace accommodations (telework, flexible schedules, and work-home balance adjustments) were not directly associated with sustainable RTW. However, an indirect association was found as workplace accommodations were associated with ergonomic adjustments (*r* = 0.35, *p* < 0.01), which in turn were associated with sustainable RTW (*r* = 0.26, *p* < 0.05) (50).

#### Qualitative findings on telework and RTW

Multiple studies highlighted remote work as a valuable accommodation for individuals with health conditions, including cancer survivors, and individuals with long-term effects of COVID-19, as it helps manage fatigue, anxiety, and cognitive impairments while facilitating RTW. However, challenges remain, such as employer reluctance, the temporary nature of accommodations, and the potential for social isolation.

For instance, Miller, Wilson (45) found that, among head and neck cancer survivors returning to work, only a minority reported supportive work experiences, such as remote work, which helped manage anxiety and fatigue (45). Similarly, Berger, Beck (48) found that adjustments like working from home were suggested as ways of improving the RTW process in cancer survivors (48). Additionally, Persoon, Buffart (46) found that for workers returning to work after stem cell transplantation for a hematologic malignancy, remote work was identified as a facilitator for returning to work (46). Stergiou-Kita, Pritlove (47) further highlighted that cancer survivors viewed remote work as a relevant accommodation, recognizing its benefits for managing fatigue and cognitive impairments (47). Similarly, many individuals with bipolar disorder highlighted the importance of occasional remote work, with the option to work from home being one of the most frequently mentioned helpful accommodations (49). Likewise, Gyllensten, Holm (44) found that telework was a key factor in facilitating RTW among individuals with the long-term effects of COVID-19. Flexible arrangements, such as remote work, were essential for many participants. Some also emphasized the importance of employer support in making this option available. While remote work provided benefits like improved recovery during the workday and a quieter, less distracting environment, concerns were raised about the temporary nature of these accommodations. Moreover, despite these advantages, some participants expressed missing the social interactions with colleagues (44).

### Findings by health condition

Individuals with acute physical illnesses (influenza-like symptoms, menstrual/pelvic pain) generally showed reduced absenteeism, as telework allowed continued work from home (39). Presenteeism effects were mixed, with some studies reporting increases (39) and others, reductions (35, 37).

Individuals with chronic physical health conditions (osteoarthritis, inflammatory arthritis, inflammatory bowel disease, long-term COVID-19, post-surgical recovery) mostly benefited from telework, which reduced absenteeism and facilitated early RTW work (32, 36, 38, 41, 42, 44). Osteoarthritis/inflammatory arthritis was an exception, showing no effect on both absenteeism and presenteeism (30).

Individuals with cancer and post-cancer treatment experienced reduced absenteeism and early RTW, as telework helped manage fatigue, cognitive impairments, and anxiety (45–48).

Individuals with chronic mental health conditions (depression, anxiety, stress disorders, bipolar disorder) also benefited from telework through reduced absenteeism and facilitated RTW when combined with flexible scheduling and supportive accommodations (36, 49).

## Discussion

### Summary of findings

This scoping review aimed to describe the impact of telework on sickness absenteeism, sickness presenteeism and RTW process among adult workers with health conditions. The included studies suggest that telework generally has positive effects on absenteeism and RTW, but presents a more complex relationship with presenteeism. Indeed, most studies indicate that telework contributes to reduced absenteeism through increased work flexibility (34, 36, 38, 39), with only one study finding no significant effect (30). Additionally, telework is consistently associated with facilitating RTW, particularly after surgery or chronic illness, by facilitating work reintegration and reducing sick leave duration (32, 38, 41, 49), with only one study finding no direct association (50). Qualitative studies highlight the role of telework in helping employees manage their health conditions more effectively, reducing unnecessary absenteeism and facilitating RTW (42–48). Regarding presenteeism, the findings are conflicting, with some studies indicating that telework increases working while sick (31, 33, 39), some suggesting it reduces working while sick (35, 37), while others report no significant effect (30) or conflicting results (40). These findings suggest that, while telework appears to reduce absenteeism and facilitate RTW, its impact on presenteeism is less consistent, which may be partially explained by contextual factors, such as health status, demographic factors, individual characteristics and organizational factors.

### Contextual factors influence on telework’s impact on presenteeism, absenteeism and return to work

The heterogeneity of findings on presenteeism and results on absenteeism and RTW may be explained by differences in health status, demographic characteristics, individual characteristics, and organizational factors across studies. These variables may have acted as mediating or confounding factors in the relationship between telework and outcomes such as presenteeism, absenteeism or RTW, as they have previously been associated with these outcomes (17, 51).

Health conditions such as back pain and psychological or mental health issues are associated with increases in both presenteeism and absenteeism (17). Telework may be a protective factor and have a greater impact on individuals with such health conditions as individuals with mental health disorders are less likely to take extended sick leave when telework options are available (36). Moreover, in cases of less severe and acute conditions such as influenza outbreaks and perhaps other infectious diseases where the risk of contagion must also be taken into account, telework can be an effective strategy to maintain productivity while limiting the spread of the disease in the workplace (39). Under these circumstances, a form of adaptive presenteeism via telework may be beneficial. Supporting this, Frolick, Wilkes (43) reported that many individuals using telework maintained productivity while ill and indicated they would have otherwise taken full sick leave if remote work had not been an option (43).

Demographic factors, such as sex, also play a role in these phenomena. Females show higher levels of presenteeism and are more likely to experience worse RTW outcomes (17, 51). The confounding impact of sex on the association between telework and presenteeism appears nuanced: while Steidelmüller, Meyer (31) reported that males had a slightly higher prevalence of sickness presenteeism, females reported a greater number of presenteeism days, suggesting potential sex differences in the intensity or duration of presenteeism during telework (31). In contrast, Takasaki (40) found no overall difference in presenteeism between full-time male and female teleworkers. A significant difference was observed only between male non-full-time teleworkers and female full-time teleworkers, indicating that the interaction between sex and telework intensity may influence presenteeism outcomes (40). Moreover, age may also play a role as younger workers may benefit more from telework, potentially due to their higher levels of presenteeism, more favorable RTW prognosis and greater technological familiarity, which may facilitate smoother adaptation to remote work environments (17, 18, 51). This could explain why one study focusing on older workers (>45 years old) found no direct association between workplace accommodations, including telework, and sustainable RTW, indicating that telework alone may be insufficient for older populations without additional supportive factors (50).

Organizational factors also contribute to the occurrence of these phenomena as presenteeism is more common among employees with high job responsibilities, workplace conflicts, low control over tasks and limited peer support (17). In contrast, longer work hours, greater job responsibilities and low supervisor support are associated with lower rates of absenteeism (17). For instance, in the context of telework, Cook and van den Hoek (35) found that when illness disclosure to a leader was included as a confounding variable, the previously significant association between telework and period pain-related presenteeism became nonsignificant. This finding suggests that organizational practices promoting openness and support may have a greater impact on reducing sickness presenteeism than telework arrangements alone (35). Similarly, Negrini, Corbière (50) found that the effectiveness of workplace accommodations like telework may depend on intermediary factors such as overall workplace health, employer and supervisor support and coworker relationships (50). Moreover, participants in the Gyllensten, Holm (44) study highlighted the crucial role of employer support in facilitating access to telework options (44). Furthermore, in cases of influenza-like symptoms, adults with access to telework were more likely to be encouraged by their employer to go home if they were at the workplace, in order to reduce contagion (39). Another organizational factor that may influence the impact of telework on work-related outcomes is the number and distribution of working hours included in telework arrangements (e.g., full-time, part-time, hybrids). For instance, two studies found an association between telework frequency and presenteeism, suggesting that the intensity of telework may influence work-related outcomes (31, 35). Work schedules can shape the extent to which employees experience the benefits or drawbacks of teleworking, such as flexibility, work-life balance, isolation and psychological distress (52, 53), which in turn can affect presenteeism, absenteeism, and RTW outcomes (17). In the present scoping review, only studies on presenteeism examined the effect of telework frequency. This highlights the need for future research to consider variations in telework schedules when examining their effects on work-related outcomes such as absenteeism or RTW. Moreover, organizational factors are not independent of country context. Although one study suggests that organizational factors explain the association between telework and presenteeism more than country differences do (31), organizational factors are nonetheless shaped by country-specific labor practices, regulations, and cultural norms. Future research should both (1) thoroughly describe the labor practices and regulations of each country studied and (2) conduct cross-country comparisons when data are drawn from multiple regions.

Finally, individual factors play an important role as psychological stress, dictated by stress tolerance, has been identified in previous research to be one of the strongest predictors of presenteeism (17). Moreover, absenteeism is associated with low organizational commitment and high job-related stress (17). Goñi-Legaz, Núñez (33) found that telework can alter employees’ behavior by increasing work during free time and presenteeism, which may, in turn, increase job stress and perpetuate the cycle of presenteeism (33). Similarly, Steidelmüller, Meyer (31) found that working during free time has a confounding effect on the relationship between telework and presenteeism (31). These findings suggest that telework may blur the work-life boundaries, which may partially explain the relationship between telework and increased presenteeism.

### Implications for policy and practice

These findings have several practical implications. First, telework can be a valuable tool to support workforce health, particularly when flexible arrangements are clearly structured and accompanied by supportive policies (44). However, without proper guidance, telework may encourage unhealthy work practices such as excessive presenteeism, as sickness presenteeism prevalence and propensity are found to increase with telework frequency (31). Therefore, employers should ensure that remote work options are implemented with integrated clear sick leave policies and training for managers to recognize signs of health-related performance issues in remote settings (33). Second, these policies should also be aligned with employee needs, particularly by ensuring that organizations are equipped and prepared to offer telework, as employees’ demand for it often exceeds its availability in many organizations (41, 49, 54). Third, employers should consider health status and conditions (e.g., reducing contagion vs. supporting RTW after surgery), demographic characteristics, individual characteristics, and organizational factors when designing telework policies as it may mediate the effect of telework on presenteeism absenteeism and RTW (8, 17, 31, 51).

### Limitation of original articles and research gaps

Existing studies on telework and work-related outcomes are not without methodological limitations that limit comparison and interpretation. First, the heterogeneity of measurement tools used for presenteeism and absenteeism in the included studies limits comparisons across studies. Moreover, some studies used unconventional methods; for example, Rousculp, Johnston (37) estimated presenteeism based on days worked with severe symptoms, using a formula that subtracted 1 day for teleworking without accounting for the total telework duration. This may have underestimated the potential of telework in reducing sickness presenteeism. Furthermore, another study assessed telework in combination with other accommodations, making it difficult to isolate the specific effect of telework or determine whether its association with ergonomic adjustments was independent (50). Additionally, most studies assessing absenteeism (6 out of 7) relied on self-reported questionnaires, which have limitations in validity and reliability, may lack depth and can be subject to inconsistent interpretation. Future research would benefit from standardized definitions and validated instruments to assess these phenomena. Second, few studies examined the longitudinal effects of telework on absenteeism, presenteeism and RTW outcomes, limiting the ability to assess causality. Third, most studies explored only one work-related outcome at a time; however, investigating the combination of these outcomes could provide more insights into their interactions. Future studies should assess the impact of telework on these work-related phenomena combined, using a longitudinal design. Moreover, incorporating mixed-method designs could provide a more complete and nuanced understanding of telework’s impact. Finally, although some studies considered health status, demographic characteristics, individual and organizational factors, their influence remains underexplored and should be incorporated into future studies and analyses to better understand the relationship between telework and presenteeism, absenteeism and RTW.

### Limitations of this scoping review

This review also has limitations. First, all studies were included regardless of the instruments or definitions they used to assess absenteeism, presenteeism or RTW, which, given the important heterogeneity across tools, limited our ability to directly compare findings. Second, this study is limited by the inclusion of only studies written or translated into English or French. This limitation may have led to the exclusion of relevant findings in other languages.

## Conclusion

This scoping review highlights the role of telework in sickness absenteeism, presenteeism, and RTW experiences among workers with health conditions. While telework appears to offer flexibility that can reduce absenteeism and facilitate RTW, its impact on presenteeism is less consistent and may even encourage working while sick if not properly supervised. Future studies should examine which policies most effectively maximize the benefits of telework while minimizing potential drawbacks, particularly regarding presenteeism and long-term health outcomes, such as an increased risk of musculoskeletal issues, burnout or social isolation (55, 56). These findings also suggest that the effectiveness of telework may depend on contextual factors, including individual health status, demographic characteristics, and organizational support. Future research should explore how these contextual factors mediate and moderate the relationship between telework and presenteeism, absenteeism, and RTW, as well as how they interact with each other.

## Data Availability

The original contributions presented in the study are included in the article/Supplementary material, further inquiries can be directed to the corresponding author.
