# Supplementary material for: The impact of telework on absenteeism, presenteeism, and return to work among workers with health conditions: a scoping review
Source: Front Public Health. 2025 Sep 9;13:1655200. doi: 10.3389/fpubh.2025.1655200 (PMC12454396; doi:10.3389/fpubh.2025.1655200)
Supplement: Supplementary file 1 [file Table_1.DOCX]

Supplementary Material

**Bibliographic search strategies**

Database: MEDLINE

Limits applied: None

| **Concepts** | **#** | **Search terms** |
| --- | --- | --- |
| Concept 1  (non-MESH terms) | 1 | AB ( "Remote work" OR Telecommut* OR Telehomework* OR Telework* OR "Home-based office" OR "Home-based telecommut*" OR "Home-based work" OR "Home work*" OR Home-work* OR Telehomework* OR "Virtual office" OR "Virtual work" OR Homme-office OR Homeoffice OR "Work8 n3 home" ) OR TI ( "Remote work" OR Telecommut* OR Telehomework* OR Telework* OR "Home-based office" OR "Home-based telecommut*" OR "Home-based work" OR "Home work*" OR Home-work* OR Telehomework* OR "Virtual office" OR "Virtual work" OR Homme-office OR Homeoffice OR "Work8 n3 home" |
| Concept 1  (MESH terms) | 2 | (MH "Teleworking") |
| Concept 1  (Combined) | 3 | 1 OR 2 |
| Concept 2  (non-MESH terms) | 4 | AB ( Return-to-work OR RTW OR "Return to work" OR "Back to work transition" OR "Job re-entry" OR "Sick leave" OR "Sick day" OR "Health-related absence" OR "Medical leave" OR "Absence due to illness" ) OR TI ( Return-to-work OR RTWOR "Return to work" OR "Back to work transition" OR "Job re-entry" OR "Sick leave" OR "Sick day" OR "Health-related absence" OR "Medical leave" OR "Absence due to illness" ) |
| Concept 2  (MESH terms) | 5 | (MH "Return to Work") OR (MH "Sick Leave") |
| Concept 2  (Combined) | 6 | 4 OR 5 |
| Concept 3  (non-MESH terms) | 7 | AB ( Absenteeism OR Non-attendance OR Absence OR Presenteeism OR "Over attendance" OR "Working while sick" ) OR TI ( Absenteeism OR Non-attendance OR Absence OR Presenteeism OR "Over attendance" OR "Working while sick" ) |
| Concept 3  (MESH terms) | 8 | (MH "Absenteeism") OR (MH "Presenteeism") |
| Concept 3  (Combined) | 9 | 7 OR 8 |
| Combination of concepts | 10 | 3 AND (6 OR 9) |

Database: CINAHL

Limits applied : None

| **Concepts** | **#** | **Search terms** |
| --- | --- | --- |
| Concept 1  (non-MESH terms) | 1 | AB ( "Remote work" OR Telecommut* OR Telehomework* OR Telework* OR "Home-based office" OR "Home-based telecommut*" OR "Home-based work" OR "Home work*" OR Home-work* OR Telehomework* OR "Virtual office" OR "Virtual work" OR Homme-office OR Homeoffice OR "Work8 n3 home" ) OR TI ( "Remote work" OR Telecommut* OR Telehomework* OR Telework* OR "Home-based office" OR "Home-based telecommut*" OR "Home-based work" OR "Home work*" OR Home-work* OR Telehomework* OR "Virtual office" OR "Virtual work" OR Homme-office OR Homeoffice OR "Work8 n3 home" |
| Concept 1  (MESH terms) | 2 | (MH "Work Environment") OR (MH "Telecommuting") |
| Concept 1  (Combined) | 3 | 1 OR 2 |
| Concept 2  (non-MESH terms) | 4 | AB ( Return-to-work OR RTW OR "Return to work" OR "Back to work transition" OR "Job re-entry" OR "Sick leave" OR "Sick day" OR "Health-related absence" OR "Medical leave" OR "Absence due to illness" ) OR TI ( Return-to-work OR RTWOR "Return to work" OR "Back to work transition" OR "Job re-entry" OR "Sick leave" OR "Sick day" OR "Health-related absence" OR "Medical leave" OR "Absence due to illness" ) |
| Concept 2  (MESH terms) | 5 | (MH "Job Re-Entry") OR (MH "Sick Leave") |
| Concept 2  (Combined) | 6 | 4 OR 5 |
| Concept 3  (non-MESH terms) | 7 | AB ( Absenteeism OR Non-attendance OR Absence OR Presenteeism OR "Over attendance" OR "Working while sick" ) OR TI ( Absenteeism OR Non-attendance OR Absence OR Presenteeism OR "Over attendance" OR "Working while sick" ) |
| Concept 3  (MESH terms) | 8 | (MH "Absenteeism") OR (MH "Presenteeism") |
| Concept 3  (Combined) | 9 | 7 OR 8 |
| Combination of concepts | 10 | 3 AND (6 OR 9) |

Database: APA PsycInfo

Limits applied : None

| **Concepts** | **#** | **Search terms** |
| --- | --- | --- |
| Concept 1  (non-MESH terms) | 1 | AB ( "Remote work" OR Telecommut* OR Telehomework* OR Telework* OR "Home-based office" OR "Home-based telecommut*" OR "Home-based work" OR "Home work*" OR Home-work* OR Telehomework* OR "Virtual office" OR "Virtual work" OR Homme-office OR Homeoffice OR "Work8 n3 home" ) OR TI ( "Remote work" OR Telecommut* OR Telehomework* OR Telework* OR "Home-based office" OR "Home-based telecommut*" OR "Home-based work" OR "Home work*" OR Home-work* OR Telehomework* OR "Virtual office" OR "Virtual work" OR Homme-office OR Homeoffice OR "Work8 n3 home" |
| Concept 1  (MESH terms) | 2 | (DE "Telecommuting") OR (DE "Teleworkers") |
| Concept 1  (Combined) | 3 | 1 OR 2 |
| Concept 2  (non-MESH terms) | 4 | AB ( Return-to-work OR RTW OR "Return to work" OR "Back to work transition" OR "Job re-entry" OR "Sick leave" OR "Sick day" OR "Health-related absence" OR "Medical leave" OR "Absence due to illness" ) OR TI ( Return-to-work OR RTWOR "Return to work" OR "Back to work transition" OR "Job re-entry" OR "Sick leave" OR "Sick day" OR "Health-related absence" OR "Medical leave" OR "Absence due to illness" ) |
| Concept 2  (MESH terms) | 5 | (DE "Reemployment") OR (DE "Employee Leave Benefits") |
| Concept 2  (Combined) | 6 | 4 OR 5 |
| Concept 3  (non-MESH terms) | 7 | AB ( Absenteeism OR Non-attendance OR Absence OR Presenteeism OR "Over attendance" OR "Working while sick" ) OR TI ( Absenteeism OR Non-attendance OR Absence OR Presenteeism OR "Over attendance" OR "Working while sick" ) |
| Concept 3  (MESH terms) | 8 | DE "Employee Absenteeism" |
| Concept 3  (Combined) | 9 | 7 OR 8 |
| Combination of concepts | 10 | 3 AND (6 OR 9) |

Database: Academic Search Complete

Limits applied : None

| **Concepts** | **#** | **Search terms** |
| --- | --- | --- |
| Concept 1  (non-MESH terms) | 1 | AB ( "Remote work" OR Telecommut* OR Telehomework* OR Telework* OR "Home-based office" OR "Home-based telecommut*" OR "Home-based work" OR "Home work*" OR Home-work* OR Telehomework* OR "Virtual office" OR "Virtual work" OR Homme-office OR Homeoffice OR "Work8 n3 home" ) OR TI ( "Remote work" OR Telecommut* OR Telehomework* OR Telework* OR "Home-based office" OR "Home-based telecommut*" OR "Home-based work" OR "Home work*" OR Home-work* OR Telehomework* OR "Virtual office" OR "Virtual work" OR Homme-office OR Homeoffice OR "Work8 n3 home" |
| Concept 1  (MESH terms) | 2 | (((DE "TELECOMMUTING") OR (DE "VIRTUAL offices")) OR (DE "VIRTUAL work")) OR (DE "HOME offices") |
| Concept 1  (Combined) | 3 | 1 OR 2 |
| Concept 2  (non-MESH terms) | 4 | AB ( Return-to-work OR RTW OR "Return to work" OR "Back to work transition" OR "Job re-entry" OR "Sick leave" OR "Sick day" OR "Health-related absence" OR "Medical leave" OR "Absence due to illness" ) OR TI ( Return-to-work OR RTWOR "Return to work" OR "Back to work transition" OR "Job re-entry" OR "Sick leave" OR "Sick day" OR "Health-related absence" OR "Medical leave" OR "Absence due to illness" ) |
| Concept 2  (MESH terms) | 5 | ((DE "RETURN to work programs") OR (DE "SICK leave")) OR (DE "JOB absenteeism") |
| Concept 2  (Combined) | 6 | 4 OR 5 |
| Concept 3  (non-MESH terms) | 7 | AB ( Absenteeism OR Non-attendance OR Absence OR Presenteeism OR "Over attendance" OR "Working while sick" ) OR TI ( Absenteeism OR Non-attendance OR Absence OR Presenteeism OR "Over attendance" OR "Working while sick" ) |
| Concept 3  (MESH terms) | 8 | (DE "JOB absenteeism") OR (DE "PRESENTEEISM (Labor)") |
| Concept 3  (Combined) | 9 | 7 OR 8 |
| Combination of concepts | 10 | 3 AND (6 OR 9) |

Database: Business Source Complete

Limits applied : None

| **Concepts** | **#** | **Search terms** |
| --- | --- | --- |
| Concept 1  (non-MESH terms) | 1 | AB ( "Remote work" OR Telecommut* OR Telehomework* OR Telework* OR "Home-based office" OR "Home-based telecommut*" OR "Home-based work" OR "Home work*" OR Home-work* OR Telehomework* OR "Virtual office" OR "Virtual work" OR Homme-office OR Homeoffice OR "Work8 n3 home" ) OR TI ( "Remote work" OR Telecommut* OR Telehomework* OR Telework* OR "Home-based office" OR "Home-based telecommut*" OR "Home-based work" OR "Home work*" OR Home-work* OR Telehomework* OR "Virtual office" OR "Virtual work" OR Homme-office OR Homeoffice OR "Work8 n3 home" |
| Concept 1  (MESH terms) | 2 | ((DE "TELECOMMUTING") OR (DE "HOME offices")) OR (DE "VIRTUAL work") |
| Concept 1  (Combined) | 3 | 1 OR 2 |
| Concept 2  (non-MESH terms) | 4 | AB ( Return-to-work OR RTW OR "Return to work" OR "Back to work transition" OR "Job re-entry" OR "Sick leave" OR "Sick day" OR "Health-related absence" OR "Medical leave" OR "Absence due to illness" ) OR TI ( Return-to-work OR RTWOR "Return to work" OR "Back to work transition" OR "Job re-entry" OR "Sick leave" OR "Sick day" OR "Health-related absence" OR "Medical leave" OR "Absence due to illness" ) |
| Concept 2  (MESH terms) | 5 | (DE "RETURN to work programs") OR (DE "SICK leave") |
| Concept 2  (Combined) | 6 | 4 OR 5 |
| Concept 3  (non-MESH terms) | 7 | AB ( Absenteeism OR Non-attendance OR Absence OR Presenteeism OR "Over attendance" OR "Working while sick" ) OR TI ( Absenteeism OR Non-attendance OR Absence OR Presenteeism OR "Over attendance" OR "Working while sick" ) |
| Concept 3  (MESH terms) | 8 | ((DE "JOB absenteeism") OR (DE "LEAVE of absence")) OR (DE "PRESENTEEISM (Labor)") |
| Concept 3  (Combined) | 9 | 7 OR 8 |
| Combination of concepts | 10 | 3 AND (6 OR 9) |

Database: Scopus

Limits applied : None

| **Concepts** | **#** | **Search terms** |
| --- | --- | --- |
| Concept 1  (non-MESH terms) | 1 | AB ( "Remote work" OR Telecommut* OR Telehomework* OR Telework* OR "Home-based office" OR "Home-based telecommut*" OR "Home-based work" OR "Home work*" OR Home-work* OR Telehomework* OR "Virtual office" OR "Virtual work" OR Homme-office OR Homeoffice OR "Work8 n3 home" ) OR TI ( "Remote work" OR Telecommut* OR Telehomework* OR Telework* OR "Home-based office" OR "Home-based telecommut*" OR "Home-based work" OR "Home work*" OR Home-work* OR Telehomework* OR "Virtual office" OR "Virtual work" OR Homme-office OR Homeoffice OR "Work8 n3 home" |
| Concept 1  (MESH terms) | 2 | NA |
| Concept 1  (Combined) | 3 | 1 OR 2 |
| Concept 2  (non-MESH terms) | 4 | AB ( Return-to-work OR RTW OR "Return to work" OR "Back to work transition" OR "Job re-entry" OR "Sick leave" OR "Sick day" OR "Health-related absence" OR "Medical leave" OR "Absence due to illness" ) OR TI ( Return-to-work OR RTWOR "Return to work" OR "Back to work transition" OR "Job re-entry" OR "Sick leave" OR "Sick day" OR "Health-related absence" OR "Medical leave" OR "Absence due to illness" ) |
| Concept 2  (MESH terms) | 5 | NA |
| Concept 2  (Combined) | 6 | 4 OR 5 |
| Concept 3  (non-MESH terms) | 7 | AB ( Absenteeism OR Non-attendance OR Absence OR Presenteeism OR "Over attendance" OR "Working while sick" ) OR TI ( Absenteeism OR Non-attendance OR Absence OR Presenteeism OR "Over attendance" OR "Working while sick" ) |
| Concept 3  (MESH terms) | 8 | NA |
| Concept 3  (Combined) | 9 | 7 OR 8 |
| Combination of concepts | 10 | 3 AND (6 OR 9) |

Database: Sociological Abstract

Limits applied : None

| **Concepts** | **#** | **Search terms** |
| --- | --- | --- |
| Concept 1  (non-MESH terms) | 1 | AB ( "Remote work" OR Telecommut* OR Telehomework* OR Telework* OR "Home-based office" OR "Home-based telecommut*" OR "Home-based work" OR "Home work*" OR Home-work* OR Telehomework* OR "Virtual office" OR "Virtual work" OR Homme-office OR Homeoffice OR "Work8 n3 home" ) OR TI ( "Remote work" OR Telecommut* OR Telehomework* OR Telework* OR "Home-based office" OR "Home-based telecommut*" OR "Home-based work" OR "Home work*" OR Home-work* OR Telehomework* OR "Virtual office" OR "Virtual work" OR Homme-office OR Homeoffice OR "Work8 n3 home" |
| Concept 1  (MESH terms) | 2 | (MAINSUBJECT.EXACT("Telecommuting") OR MAINSUBJECT.EXACT("Work at home")) OR (MAINSUBJECT.EXACT("Telecommuting") OR MAINSUBJECT.EXACT("Work at home")) |
| Concept 1  (Combined) | 3 | 1 OR 2 |
| Concept 2  (non-MESH terms) | 4 | AB ( Return-to-work OR RTW OR "Return to work" OR "Back to work transition" OR "Job re-entry" OR "Sick leave" OR "Sick day" OR "Health-related absence" OR "Medical leave" OR "Absence due to illness" ) OR TI ( Return-to-work OR RTWOR "Return to work" OR "Back to work transition" OR "Job re-entry" OR "Sick leave" OR "Sick day" OR "Health-related absence" OR "Medical leave" OR "Absence due to illness" ) |
| Concept 2  (MESH terms) | 5 | NA |
| Concept 2  (Combined) | 6 | 4 OR 5 |
| Concept 3  (non-MESH terms) | 7 | AB ( Absenteeism OR Non-attendance OR Absence OR Presenteeism OR "Over attendance" OR "Working while sick" ) OR TI ( Absenteeism OR Non-attendance OR Absence OR Presenteeism OR "Over attendance" OR "Working while sick" ) |
| Concept 3  (MESH terms) | 8 | MAINSUBJECT.EXACT("Absenteeism") OR MAINSUBJECT.EXACT("Absenteeism") |
| Concept 3  (Combined) | 9 | 7 OR 8 |
| Combination of concepts | 10 | 3 AND (6 OR 9) |

Database: ABI/FORM Global

Limits applied : None

| **Concepts** | **#** | **Search terms** |
| --- | --- | --- |
| Concept 1  (non-MESH terms) | 1 | AB ( "Remote work" OR Telecommut* OR Telehomework* OR Telework* OR "Home-based office" OR "Home-based telecommut*" OR "Home-based work" OR "Home work*" OR Home-work* OR Telehomework* OR "Virtual office" OR "Virtual work" OR Homme-office OR Homeoffice OR "Work8 n3 home" ) OR TI ( "Remote work" OR Telecommut* OR Telehomework* OR Telework* OR "Home-based office" OR "Home-based telecommut*" OR "Home-based work" OR "Home work*" OR Home-work* OR Telehomework* OR "Virtual office" OR "Virtual work" OR Homme-office OR Homeoffice OR "Work8 n3 home" |
| Concept 1  (MESH terms) | 2 | MAINSUBJECT.EXACT("Virtual offices") OR MAINSUBJECT.EXACT("Telecommuting") OR MAINSUBJECT.EXACT("Work at home") |
| Concept 1  (Combined) | 3 | 1 OR 2 |
| Concept 2  (non-MESH terms) | 4 | AB ( Return-to-work OR RTW OR "Return to work" OR "Back to work transition" OR "Job re-entry" OR "Sick leave" OR "Sick day" OR "Health-related absence" OR "Medical leave" OR "Absence due to illness" ) OR TI ( Return-to-work OR RTWOR "Return to work" OR "Back to work transition" OR "Job re-entry" OR "Sick leave" OR "Sick day" OR "Health-related absence" OR "Medical leave" OR "Absence due to illness" )virtual |
| Concept 2  (MESH terms) | 5 | (MAINSUBJECT.EXACT("Return to work programs") OR MAINSUBJECT.EXACT("Sick leave")) OR (MAINSUBJECT.EXACT("Return to work programs") OR MAINSUBJECT.EXACT("Sick leave")) |
| Concept 2  (Combined) | 6 | 4 OR 5 |
| Concept 3  (non-MESH terms) | 7 | AB ( Absenteeism OR Non-attendance OR Absence OR Presenteeism OR "Over attendance" OR "Working while sick" ) OR TI ( Absenteeism OR Non-attendance OR Absence OR Presenteeism OR "Over attendance" OR "Working while sick" ) |
| Concept 3  (MESH terms) | 8 | (MAINSUBJECT.EXACT("Absenteeism") OR MAINSUBJECT.EXACT("Leaves of absence")) OR (MAINSUBJECT.EXACT("Absenteeism") OR MAINSUBJECT.EXACT("Leaves of absence")) |
| Concept 3  (Combined) | 9 | 7 OR 8 |
| Combination of concepts | 10 | 3 AND (6 OR 9) |

**Supplementary material 1.** Bibliographic search strategies
