# Supplementary material for: The impact of telework on absenteeism, presenteeism, and return to work among workers with health conditions: a scoping review
Source: Front Public Health. 2025 Sep 9;13:1655200. doi: 10.3389/fpubh.2025.1655200 (PMC12454396; doi:10.3389/fpubh.2025.1655200)
Supplement: Supplementary file 2 [file Table_2.DOCX]

| Author, year | Objectives (Study design) | Variable measured (presenteeism, absenteeism, return to work and telework) | N | Health status | Demographic factors | Individual characteristics | Organizational factors |
| --- | --- | --- | --- | --- | --- | --- | --- |
| Berger, I. (2020) | To examine cancer survivors’ perspectives on supports needed when returning to work or staying in the workforce and to explore personal and employment factors that influence the return-to-work process.  (Qualitative study) | **Return to work:**  Participants were asked questions about their overall experience when returning to work or staying at work following their cancer diagnosis. | 15 | Cancer survivors. | **Age:**  18 years of age or older.  **Sex (n):**  - Female: 13.  - Male: 2. | **Psychological stress:** Participants discussed what it meant to “feel ready” to return to work, and how difficult it was to ever feel completely ready to go back. Feeling ready to return involves many different elements, such as emotional, mental, and physical readiness. For some, these hardships were influenced by having to lead their own return to work plan, which was stressful and uncomfortable. | **Manager’s support:**  Participants were instructed to only communicate with insurance and were not allowed to have contact with their managers, which they would have preferred. This experience was confusing and stressful for many participants, as it left them with a lot of uncertainty about their ability to return to work and whether they were communicating appropriately.  **Co-workers’support:**  Despite feeling stigma sometimes at work, some participants felt comfortable discussing their experiences openly with their coworkers in order to ease the transition back into work.  **Working hours:**  Survivors requested less working hours in order to manage their symptoms. |
| Gignac, M. A. (2015) | To examine the availability, need, and use of workplace policies for workers with osteoarthritis and  inflammatory arthritis and their association with employment outcomes.  (Prospective cohort study) | **Presenteeism:**  The 12-item Workplace Activity Limitations Scale measured arthritis-related activity limitations at work. Productivity loss was measured by asking participants whether arthritis or its treatment resulted in being less productive at work in the past 6 months (where 1 = not at all and 5 = a great deal). | 219 | - Diagnosed with osteoarthritis: 48.9%.  - Diagnosed with inflammatory arthritis: 51.1%. | **Age:**  53.0 years (SD = 8.1)  **Sex (%):**  - Women: 72.8.  - Men: 27.2.  **Marital status n (%):**  - Married: 116 (53.5)  - Single, divorced, widowed, separated: 101 (46.5) | N/A | **Job control:**  Ten items asked about job control over work tasks, pace, and scheduling (where 1= very little and 5= very much):  - Job control (range 0–50) (mean +/- SD): 31.4 +/- 13.4.  Bivariate analyses revealed that those who had greater job control were more likely to report needing >=2 benefits/accommodations (p <0.20) and were more likely to lead to using >/=2 benefits/accommodations (p <0.20).  **Manager’s support:**  Nearly 62% of respondents had disclosed their arthritis to their manager.  **Working hours:**  Weekly working hours mean+/- SD: 34.6 +/- 11.9  **Working industry**  38.1% worked in health, teaching, sciences, or the arts, 31.2% in business, finance, or administration, 21.4% in sales or services, and 9.3% in trades, transportation, or equipment operation. |
| Steidelmuller, C. (2020) | To investigate the relationship between home-based telework and presenteeism.  (Cross-sectional design) | **Presenteeism:**  Three different approaches were used to measure sickness presenteeism.  The first measure asks, "In the past 12 months, did you work while sick?" (yes=1, no=0). The second measured the total number of days worked while sick (presenteeism). The third is the sickness presenteeism propensity, calculated as the ratio of presenteeism days to the total days of sickness (presenteeism + sickness absence), ranging from 0 (only sickness absence) to 1 (only presenteeism).  **Telework:**  Telework was defined by the question: "How often have you worked in each location during the last 12 months - Your own home?" with a response scale taking the frequency into account: daily, several times a week, several times a month, less often, and never. | 25,465 | **Self-rated health status:**  26.5% of participants rated their health as very good, 53.6% as good, 17.8% as fair, 1.9% as bad, and 0.2% as very bad. | **Age (%):**  - 15 to 29 =17.5  - 30 to 39 =25.0  - 40 to 49 =27.9  - 50 to 65 =29.7  **Sex:**  - Male: 50.7%  - Female: 49.3% | N/A | **Working hours (%):**  - Part-time 21.6  - Full-time 78.4  **Working industry**  1.5% worked in agriculture, 18.9% in industry, 6.0% in construction, 18.5% in commerce and hospitality, 6.2% in transport, 3.8% in financial services, 6.8% in public administration and defense, 9.6% in education, 12.6% in health, and 16.1% in other services |
| Kangas (2024) | To evaluate how the coordinated return to work model affects return to work (RTW) after primary hip (THA) or total/unicondylar knee (KJA) arthroplasty, and what kind of actions have been made in occupational health care and in the workplace for supporting RTW. To explores patient and work-dependent factors influencing early RTW, and patients’ experiences of the CRTW model.  (Prospective Cohort study) | **Return to work:**  Time to RTW was determined as the number of days between the operation day and the self-reported day of returning to work. Returning to full-time and part-time work were both defined as successful RTW. | 209 | - **Underwent THA (n):** 94  - **Underwent KJA (n):** 115 | **Age:**  25 to 62 years  **Sex (n):**  129 females.  80 males | **Educational levels:**  22 low education level, 107 intermediated, 75 high and 5 missing. | N/A |
| Goni-Legaz (2023) | To investigate how home-based telework (HBT) affects job stress.  (Cross-sectional design) | **Presenteeism:**  The number of days of presenteeism was measured using a question related to the number of working days the respondent worked when they were sick in the last year in a free-response format.  **Absenteeism:**  The number of days of absenteeism was measured using a question of how many days in total over the past 12 months the employees were absent from work due to sick leave.  **Telework:**  1. Home-based teleworkers: employees working from home regularly (at least several times a month).  2. High-mobile teleworkers: employees working in several places regularly, with a high level of mobility.  3. Occasional teleworkers: employees occasionally working in one or more places outside the employer’s premises.  4. Face-to-face employees: employees work always at the employer’s premises. | 5,244 | N/A | N/A | **Psychological stress:**  Job stress (n) = 3.080:  - Never 7.97%  - Rarely 17.30%  - Sometimes 43.48%  - Most of the time 18.57%  - Always 12.68%  Results suggest the effect of HBT on job stress was mediated (to a large extent) by working in free time and presenteeism. | **Working hours:**  Working in free time (n)= 1.857  - Never: 49.18%  - Less often: 26.96%  - Several times a month: 15.05%  - Several times a week: 6.64%  - Daily: 2.17%  **Working Status**  1097 doing home base telework, 1789 High-mobile teleworkers, 1205 Occasional mobile teleworkers, 7333 Always at the employer’s premises (face-to-face) (Total before eliminating missing data) |
| Borge (2023) | To investigate the main effects of office design and access to telework from home (TWFH) on self-certified sickness absence and the moderating effects of access to TWFH on the relationship between office design and self-certified sickness absence.  (Cross-sectional design) | **Absenteeism:**  Measured based on two questions: In the last 12 months (1) “Have you had self-certified sickness absence in the last 12 months?”, and (2) “How many times/episodes with self-certified sickness absence have you had in the last 12 months?”.  **Telework:**  Access to TWFH was measured by a dichotomous variable based question, “Can you work from home in your current job?”. | 4,329 | N/A | **Age (SD):**  Mean age: 45.97 (11.13)  **Sex n (%):**  - Female: 2,008 (46)  - Male: 2,321 (54) | **Educational levels:**  24% (n = 1,018) had a university/college education of five years or more, 39% (n = 1,685) had one to four years of university/college education, 26% (n = 1,139) completed upper secondary school, and 11% (n = 479) had primary or lower secondary education. Data on education were missing for eight participants. | **Access to telework from home n (%):**  Yes: 2,943 (68%)  No: 1,386 (32%).  **Leadership responsibility** **n (%):**  - No: 2425 (58)  - Yes: 1768 (42)  - Missing: 136  **Working hours:**  Time spent doing office work: n (%):  - ≥50%: 3262 (75)  - <50%: 1067 (25) |
| Cook (2023) | To investigate the associations between period pain and health-related and work-related factors, with the latter focus on the role of supervisors or managers and remote work.  (Cross-sectional design) | **Presenteeism:**  Abridged version of the Hagerbaumer presenteeism scale.  **Telework:**  Telework was assessed with one item: “On average, how many days per week have you been working remotely in the past six months?”. | 668 | - n=236 (35.33%) participants had no underlying diagnosis (primary dysmenorrhea).  - n=383 stated having a medical diagnosis of endometriosis.  - n=43 had polycystic ovary syndrome  - n=48 adenomyosis.  - n=29 uterine myoma  - n=2 pelvic inflammatory disease  - n=17 stated having a different underlying medical diagnosis. | **Age:**  29.89 years (SD 6.04), ranging from 18 to 53  **Sex and gender n (%)**  - Identified as female: 659.  - Identified as non-binary: 7 (1.04%).  - Identified as male: 1.  - Checked the “no answer”: 1. | N/A | **Manager’s support:**  - The more information about the health condition was disclosed to their manager, the less likely participants were to engage in period pain presenteeism.  - Results showed that employees disclosed more if they had a good relationship with their leader and when their leader was not male.  - Leader-member exchange was negatively and significantly correlated with having a male leader, r=- 0.09, p=0.02.  **Working status**  About two thirds stated having a permanent contract (75.55%). Participants worked in various industries, with the largest group working in health care (N=154, 23.05%).  **Working hours, mean (SD):**  **-** Days/week remote work: 1.90 (2.00)  - Hours/week: 35.56 (6.46)  - Working hours per week correlated positively with having a male leader, r=0.09, p =0.03, and negatively with working in a more female-dominated industry, r=-0.15, p <0.01. |
| Helgesson (2023) | To investigate the relationship between psychosocial and organizational workplace factors and sickness absence three years after participation in the Swedish Work Environment Survey, and if there are variations regarding these relationships in different common mental disorder diagnoses.  (Cross-sectional design) | **Absenteeism:**  Sickness absence in this study covers sick-leave periods of 15 days or more. Moreover, two days of half-time sickness absence was counted as one net day of sickness absence.  **Telework:**  Measured by asking, “How much of your normal working time do you usually work at home”. | 3,795 | All participants are with different common mental disorder diagnoses. | **Age n (%):**  - 16 to 25: 172 (4.5)  - 26 to 35: 568 (15.0)  - 36 to 45: 957 (25.2)  - 46 to 55: 1190 (31.4)  - 56 to 64: 908 (23.9)  **Sex, n (%):**  - Women: 2596 (68.4)  - Men: 1199 (31.6) | **Psychological stress:**  - Job strain was defined following the work demand-control model with several variables serving as proxies.  - Job strain seemed most detrimental to individuals with depression.  **Educational level:**  15.3% had a low educational level (elementary school), 49.1% had a medium level (upper secondary school), and 35.6% had a high level (university). | **Control over tasks**  Experiencing low control over work (RR=1.16 95% CI: 1.01–1.35) and experiencing high job strain (RR=1.25 95% CI: 1.04–1.49) were related to an increased risk of long-term sickness absence.  **Manager and colleagues’ support**  No significant association between social support at work and having at least one period over 14 days of sickness absence during the follow-up.  **Working hours**  Having no flexibility regarding working hours (RR: 1.25, 95% CI: 1.08–1.45) and having no possibility to working from home (RR: 1.37, 95% CI: 1.13–1.66) was connected to having a higher risk of experiencing a spell of over 14 days of sickness absence in the follow-up period. |
| Rousculp (2010) | To examine the impact of various flexible sick leave policies (FSLPs) on workplace attendance of employees with self-reported “severe” influenza-like-illness (ILI) symptoms.  (Prospective observational cohort study) | **Presenteeism:**  The number of days that an employee attended work while their symptoms were most severe  **Absenteeism:**  The number of days absent from work because of the ILI symptoms  **Telework:**  If participants worked from home instead of their typical worksite because of their ILI symptoms. | 793 | 1,104 illness months with reported ILI symptoms. | **Age (yr), mean (SD):** 40.7 (7.1)  **Sex n (%):**  Female: 282 (35.6) | **Children under custody:** Primary caregiver to children, n (%): 356 (44.9) | **Workplace factors**  34.6% with adjustable hours, 35.4% with paid time off, and 10.3% with work-from-home options. 49% worked in precision production, crafts, or laborer roles, and 44.9% were primary caregivers. |
| Van Doninck (2022) | To analyze whether job-related features are associated with time off work following arthroscopic partial meniscectomy.  (Retrospective study) | **Absenteeism:**  Sick leave was defined as time off from previous employment.  **Telework:**  Telework was defined as a work  arrangement that allowed patients to perform the same or alternative work at home. | 63 | - Medial meniscus affected:  51% (n=81).  - Lateral meniscus affected: 9% (n=14).  - Medial + lateral meniscus affected: 3% (n=5) | **- Age, mean (SD)**: 45 (9,19)  **- Sex:**  Male: 70% | N/A | **Control over tasks**  - Telework, white collar and self-employment were significantly associated with early return to work (p ≤0,001).  - Self-employed patients had the lowest sick leave with 7 days (95% CI: -3,81-17,01).  **Working status**  Salaried (92%)  Blue-collar workers (70 %). |
| Negrini, A. (2020) | To identify the determinants of a sustainable return to work (RTW) for workers aged 45 and over who have suffered from a work-related injury of a psychological or physical nature.  (Research report, mixed-method design, qualitative longitudinal) | **Return-to-work duration:**  The prediction of sustainable return to work was assessed using the proxy variable measuring the duration of sustainable return to work (distance in months between the date of return to work and the date of T2 (6 months after the first round of questionnaires). | 65 | RTW n (%) after:  - **Psychological injury:** 34 (52)  **- Physical injury:** 31 (48) | **Age: mean (SD):**  52 (5.2), ranging from 45 to 67  **Sex:**  Women n (%): 42 (65).  **Marital status n (%):** the majority of participants are married, in a common-law de facto union or as a couple: 39 (60) | **Children under custody:**  Having one or more children, n (%): 26 (81).  **Education level:**  Most participants had obtained a college diploma (n = 16; 25%). | **Colleagues’ support**  Participants with physical injury receive more support from their colleagues than participants with psychological injury.  **Working hours**  Participants work an average of 38 hours a week (SD = 7.21; min = 15 hours; max = 55 hours). |
| Coenen (2018) | To improve both the well-being of the patient as well as his/her employer, and to contribute toward a reduction in absenteeism by focusing on the workability of individuals with inflammatory bowel diseases.  (Qualitative study) | **Telework and absenteeism:**  The patients completed the study specific timesheet at the end of every month (worked days from office, worked days from home, absences because of sick leave). | 14 | Ulcerative colitis: 57%. | **Age:**  Older than 18 years  **Sex (%):**  Female: 71 | **Psychological stress:**  Removing work-related stress factors resulted in employees feeling much  more at ease. Concretely, this led to an absence of sick leave for more than 50% of the included patients.  By teleworking, patients could determine work rhythm by themselves (take a rest when needed, start working when ready, and have a toilet nearby). This resulted in less stress and had a positive impact on the overall work–life balance of the patients. | **Control over tasks:**  The employee can start and stop the working day as needed and incorporate moments of rest. However, the employer decides at which time points the employee is required to be present at the workplace.  **Manager’s support:**  - More openness was created between the employee and the employer and that the task package of the employee was reviewed critically.  - There is still a barrier for employers to keep the chronically ill at work and to reintegrate them. |
| Frolick (1993) | To explore factors that an organization should consider before implementing a telecommuting program. (qualitative study) | N/A | 45 | N/A | N/A | N/A | **Working hours:**  - An average time spent in telework programs: 2.3 years.  - Maximum work time from home: 4 days per week.  **Working status:**  10 telework managers, 10 telework administrators, and 25 teleworkers |
| Gyllensten, K. (2023) | To identify workplace factors that promote and hinder work ability and return to work among individuals with long-term effects of COVID-19.  (Qualitative study) | **Presenteeism and absenteeism:**  Questionnaires for assessing work ability score and sickness absence were conducted.  **Return to work:**  Semi-structured focus group interviews. Questions: ‘What at work has made it more difficult/ easy to return to work?’ ‘What factors have had a positive/negative effect on your work ability?’Return to work | 19 | **Long-term symptoms of COVID-19: median number of months since contracting COVID-19: n (mean):**  18 (15), range of 44 to 22 months). | **Age:**  ranged between 39-63 (mean: 54)  **Sex n (%):**  - Female: 13 (68.4)  - Male: 6 | N/A | **Manager’s support:**  Good communication and sufficient support from the managers were viewed as almost necessary for being able to have a reasonable work situation while suffering from long-term effects of COVID-19:  - Managers had suggested that some tasks should be removed in order to decrease the workload.  - There was a lack of understanding from the employer.  - Some participant wished that they would show empathy.  **Colleagues’ support:**  - In some instances, colleagues had volunteered to take over the more demanding work tasks in order to decrease the work demands for the participants.  - Others felt that their colleagues silently were watching out for them to make sure that they were feeling all right, and this provided a sense of being cared for.  **Working hours:**  - Various forms of adjustments were helpful: such as telework, having flexible working hours  and adjusted work tasks.  -All participants were working at least 50% at the time of the recruitment. |
| Miller (2023) | To understand experiences, establish head and neck cancer survivor priorities for practice, and situate knowledge within context.  (Qualitative study) | **Return to work:**  Returning to work signified recovery, and the resumption of « normality » and pre-diagnosis life. | 13 | Head and neck cancer survivor (time since ranged between 3 months and 20 years; median 24 months). | **Age (mean):**  54, ranging between 39 and 63 years old.  **Sex (%):**  Female: 38.5. | **Job satisfaction:**  Regular communication with managers and colleagues enhanced workplace satisfaction. | **Manager and colleagues’ support:**  A new job, or returning to supportive workplaces, motivated recovery following treatment, for both social and work achievement reasons. Negative work interactions, and poor communication from managers and colleagues, exacerbated poor mental health and eroded confidence levels on returning to work. |
| Persoon, S. (2018) | To identify hematopoietic stem cell transplantation (HSCT) survivors’ (return to) work (RTW) perceptions, barriers to and facilitators of RTW, and possible solutions to improve RTW.  (Qualitative study) | **Return to work:**  Face-to-face semi-structured interviews on: perceived barriers to and facilitators of RTW, and possible solutions to improve RTW. | 15 | Patients treated with stem cell transplantation for a hematologic malignancy (average 30 months (range 15-57) post-HSCT). | **Age (median):**  48, ranging between 30-59 years old.  **Sex (%):**  Female: 47. | **Children under custody**  Taking care of household tasks and/or children, and commuting were mentioned as barriers. | **Working hours:**  Working at least 12 h per week in the year prior to the HSCT.  **Working status:**  Before diagnosed: 80% permanent employment, 13% self-employed, 7% temporary employment contract.  **Conflicts, managers and colleagues’ support:**  - Insufficient recognition from colleagues, no understanding of the (long-term) consequences of HSCT, disagreement about which tasks the patients were entitled to do.  - Work relations provided support and understanding and a safe place to return to.  - Adequate RTW support from the supervisor, occupational physician, and human resource management was also identified as facilitators. |
| Stergiou-Kita (2015) | To gain an understanding of the process elements necessary to identifying and successfully implementing accommodations following cancer by targeting the experiences of three stakeholder groups involved in the request and provision of workplace accommodations: (1) cancer survivors, (2) their health/vocational rehabilitation service providers, and (3) employer representatives.  (Qualitative study) | **Return to work:**  Semi-structured interviews about their experiences of working or returning to work and/or accommodations they requested and were provided/not provided at the workplace. | 40 | **16 cancer survivors (n):**  - Breast cancer: 6  - Leukemia: 2  - Bladder cancer: 1  - Hodgkin’s lymphoma: 1  - Kidney cancer: 1  - Myeloma: 1  - Ovarian and uterine cancer: 1  - Retroperitoneal liposarcoma: 1  - Sarcoma: 1  - Thyloma: 1 | **Age, mean (SD):** 48.19 (9.03)  **Sex (%):**  Female: 75 | N/A | **Responsibility:**  Jobs which involved high levels of responsibilities or high-performance expectations were viewed as less accommodating based on established productivity expectations.  **Manager and colleagues’ support:**  Assistance from a co-worker, a volunteer, or a paid assistant was also viewed as relevant for ensuring essential tasks could be completed. Stakeholders referenced the importance of pre-illness relationships with supervisors or co-workers in the workplace. |
| Varekamp (2010) | To identify the practical and psychosocial barriers recognized by employees with chronic disease who experience work-related problems and to examine preferred work accommodations.  (Quantitative study) | **Telework:**  Work accommodations were measured with work accommodations list of Kremer et al. (2006).  A baseline mail questionnaire and an interview question were asked about employees’ work accommodations. | 122 | **Chronic diseases: mean** **(SD):**  - Diseases of the musculoskeletal system and connective tissue: 30 (25)  - Diseases of the nervous system: 28 (23)  - Diseases of the digestive system: 25 (21)  - Endocrine, nutritional and metabolic diseases: 10 (8)  - Neoplasms: 6 (5)  - Diseases of the respiratory system: 5 (4)  - Diseases of the circulatory system: 4 (3)  - Other diseases: 14 (12)  - One or more additional chronic disease: 54 (44)  - Disease duration in years: 10.2 (9.5) | **Age, mean (%):**  45.6 (8.7)  **Sex (%):**  Female: 91  **Marital status: n (%):**  Living alone (no partner, children  or parents): 34 (28) | **Job satisfaction:**  (0–100): study population: mean (SD) = 22.0 (29.1) vs Dutch working population: mean (SD) = 12.7 (18.8)*  *Quality of work scales: a higher score means a more unfavorable situation.*  **P , 0.001.* | **Control over tasks and working hours:**  - Job autonomy (0–100): study population, mean (SD)= 44.1 (20.0) vs Dutch working population, mean (SD) = 41.7 (19.4)  - 90% of the respondents stated that they preferred (more) work accommodations. Working fewer hours and telework were preferred accommodations, as well as a slower work pace, control over the planning of tasks, alternative working hours, less tasks, extra training, assistance from others and a better workplace climate.  **Manager and colleagues’ support:**  - Social relationship, supervisor (0–100): study population: mean (SD)= 25.2 (18.5) vs Dutch working population: mean (SD) = 23.0 (16.9)  - Social relationship, colleagues (0–100): study population: mean (SD)= 27.0 (14.3) vs Dutch working population: mean (SD) = 22.2 (13.9)*  *Quality of work scales: a higher score means a more unfavorable situation.*  **p= 0.001.*  - Questions asked: Due to my disease, I experience problems to contact with supervisors or line managers (%): - No: 52; Yes slightly: 34; Yes, severely: 14  Due to my disease, I experience  problems to contact with colleagues (%): No: 53; Yes, slightly: 41; Yes, severely: 7 |
| Tremblay (2011) | To identify job characteristics and workplace policies conducive to the job success of individuals with bipolar disorder, and to examine the interactions between employers and bipolar employees regarding requested workplace accommodations.  (Cross-sectional, descriptive and qualitative) | **Presenteeism:**  Being present at work but performing poorly due to illness.  **Telework:**  Each nonworker marked one of four boxes indicating if a policy was unimportant, helpful, important, or necessary for them to work in a paid job. | 39 | - Mean age of illness onset: 22 years.  - 43.6% (n = 17) are diagnosed with Bipolar I disorder.  - 65.7% (n = 23) experience rapid or ultra-rapid cycling.  - 46.0% (n = 17) have experienced a psychotic episode.  - 92.3% (n = 36) are using medication. | - **Age (mean):** 47  **- Sex: n (%):**  Female: 23 (59) | **Education level:**  56.4% (n = 22) have completed college. 26 workers | **Working status:**  26 workers, 13 non-workers.  **Control over work:**  - Bipolar workers appear to have a high level of autonomy on the job.  - Decision without supervision: 96%.  - Set your own tasks: 92%.  - The majority of non-workers were more likely to take the job if they were free to set their own tasks, priorities or goals (82%) and were free to make decisions without supervision (73%).  **Responsibility:**  - Of the nonworkers, 58% would not take a job or would be less likely to take a job if it required “making decisions which affect other people or the image or financial resources of the employer,” or if they were responsible for work outcomes of others.  - Responsibility for the safety of other workers was rated negatively by 42%.  - 73% of the workers make decisions that affect people or their employers, 58% are responsible for the work outcomes of others, and 46% are responsible for the safety of others.  **Group-work conflicts:**  42% of the non-workers negatively rated working in teams (80% of those employed work in teams).  **Disclosure to managers about bipolar diagnosis (n):**  - For workers: 15 *(3 said they were better off, one said he or she was worse off and 11 said there was no difference)*  - For non-workers: 9 *(5 said they were worse off, one said he or she was better off, and 3 said there was no difference)*  **Managers’ support:**  - In several cases, employers gave the employee access to the workplace after hours.  - Relieving job pressure, job reassignment and better lighting were also noted as helpful changes.  **Working hours:**  Schedule flexibility was one of the most commonly reported advantageous work attributes. |
| Takasaki (2024) | **(Primary aim)** To investigate whether a difference in presenteeism exists among four groups of male and female teleworkers with pain and male and female non-teleworkers with pain.  **(Secondary aim)** To create a model with presenteeism as the dependent variable and the degree of the potentially relevant biopsychosocial factors for each group as the independent variables. | **Presenteeism:**  The loss of work productivity or ability because of health issues in workers, which can be measured subjectively.  **Telework:**  In this study, teleworkers were defined as those who teleworked >70% of the time at home, while others were defined as non-full teleworkers. | 2,700 | The participants were asked if they were diagnosed with the following 24 comorbidities, reported with a binary scale (0=no, 1=yes): (1) allergy, (2) anxiety, (3) arthritis, (4) asthma, (5) bladder/urinary, (6) chronic bronchitis/emphysema, (7) congestive heart failure, (8) chronic obstructive pulmonary disease, (9) coronary heart disease, (10) depression, (11) diabetes, (12) chronic fatigue syndrome, (13) gastroesophageal reflux disease, (14) headache, (15) hypertension, (16) irritable bowel, (17) migraine, (18) obesity, (19) osteoporosis, (20) skin cancer, (21) other cancer, (22) chronic pain, (23) sleeping problem, and (24) ulcer.  *Refer to the article for detailed information.* | **Age (mean):**  - Men non-full teleworkers with pain: 49.1  - Men full or almost full teleworkers with pain: 48.8  - Female non-full teleworkers with pain: 43.5  - Female full or almost full teleworkers with pain: 42.8  **Sex (n):**  - Female: 1,350  - Male: 1,350 | The nine factors of work-related stress and four work environmental factors were investigated using the Brief Job Satisfaction Questionnaire (BJSQ) (0–4).  **Psychological stress:**  - Men non-full teleworkers with pain: 2.2 (0.9)  - Men full or almost full teleworkers with pain: 2.1 (0.9)  - Female non-full teleworkers with pain: 1.8 (1.3)  - Female full or almost full teleworkers with pain: 1.8 (1.3)  **Job satisfaction:**  - Men non-full teleworkers with pain: 2.4 (1.1)  - Men full or almost full teleworkers with pain: 2.4 (1.0)  - Female non-full teleworkers with pain: 2.5 (1.1)  - Female full or almost full teleworkers with pain: 2.5 (1.1)  **Job commitment (“worth working for”):**  - Men non-full teleworkers with pain: 2.3 (1.1)  - Men full or almost full teleworkers with pain: 2.2 (1.1)  - Female non-full teleworkers with pain: 2.4 (1.1)  - Female full or almost full teleworkers with pain: 2.3 (1.2) | **Work status:**  Male non-full teleworkers with pain: n=1,068  Male full or almost full teeworkers with pain: n=282  Female non-full teleworkers with pain: n=1,043 Female full or almost full teleworkers with pain: n=307  **Control over work (BJSQ):**  - Men non-full teleworkers with pain: 1.6 (1.1  - Men full or almost full teleworkers with pain: 1.5 (1.0)  - Female non-full teleworkers with pain: 1.6 (1.0)  - Female full or almost full teleworkers with pain: 1.5 (0.9)  ***Manager* and colleagues’ support (BJSQ):**  - Men non-full teleworkers with pain: *2.4 (1.2)*, 2.6 (1.1)  - Men full or almost full teleworkers with pain: *2.4 (1.1)*, 2.6 (1.0)  - Female non-full teleworkers with pain: *2.1 (1.1)*, 2.7 (1.1)  - Female full or almost full teleworkers with pain: *2.1 (1.1)*, 2.7 (1.1)  **Overworking hours (h/week):**  - Men non-full teleworkers with pain: 6.6 (7.4)  - Men full or almost full teleworkers with pain: 5.2 (6.5)  - Female non-full teleworkers with pain: 2.9 (5.2)  - Female full or almost full teleworkers with pain: 3.1 (5.9) |
| Ahmed (2020) | To assess the association between access to telework and paid leave benefits and short-term work attendance in employed adults during a medically attended Acute Respiratory Illness (ARI) or influenza episode. | **Telework:**  Habitual teleworkers = regularly worked from home >/=1 hour in a typical week. | 1,374 | Patients seeking care for an ARI with cough within 7 days of illness onset during November 1, 2017–April 19, 2018 (the 2017–18 influenza season), at outpatient facilities affiliated with sites participating in the US Influenza Vaccine Effectiveness Network.  Thirty-six percent of participants had laboratory-confirmed influenza. | **Age (mean):**  42  **Sex (%):**  Female: 64  **Ethnicity (%):**  Non-Hispanic white: 82 | N/A | **Working hours:**  Part-time workers were those  working >/=20 but <35 hours; full-time was defined as >/=35 hours/week  Among persons with access to telework, the median  hours usually teleworked was 8 hours/week (5th, 95th percentile: 2, 30 hours/week). |

**Supplementary Material 2.** Extraction table presents study authors, year, study design, sample size, study objectives, variables related to presenteeism, absenteeism, return to work, telework and contextual factor. Contextual factors are organized into categories based on the framework proposed by Gosselin et al. (2013).
